# Convergence of circuit dysfunction in ASD: a common bridge between diverse genetic and environmental risk factors and common clinical electrophysiology

**DOI:** 10.3389/fncel.2014.00414

**Published:** 2014-12-08

**Authors:** Russell G. Port, Michael J. Gandal, Timothy P. L. Roberts, Steven J. Siegel, Gregory C. Carlson

**Affiliations:** ^1^Department of Psychiatry, Perelman School of Medicine, University of PennsylvaniaPhiladelphia, PA, USA; ^2^Semel Institute for Neuroscience and Human Behavior, University of California at Los AngelesLos Angeles, CA, USA; ^3^Bioengineering Graduate Group, University of PennsylvaniaPhiladelphia, PA, USA; ^4^Department of Radiology, Lurie Family Foundations MEG Imaging Center, The Children’s Hospital of PhiladelphiaPhiladelphia, PA, USA

**Keywords:** ASD, circuit, gamma, VSDi, translational, EEG, MEG, neurophysiology

## Abstract

Most recent estimates indicate that 1 in 68 children are affected by an autism spectrum disorder (ASD). Though decades of research have uncovered much about these disorders, the pathological mechanism remains unknown. Hampering efforts is the seeming inability to integrate findings over the micro to macro scales of study, from changes in molecular, synaptic and cellular function to large-scale brain dysfunction impacting sensory, communicative, motor and cognitive activity. In this review, we describe how studies focusing on neuronal circuit function provide unique context for identifying common neurobiological disease mechanisms of ASD. We discuss how recent EEG and MEG studies in subjects with ASD have repeatedly shown alterations in ensemble population recordings (both in simple evoked related potential latencies and specific frequency subcomponents). Because these disease-associated electrophysiological abnormalities have been recapitulated in rodent models, studying circuit differences in these models may provide access to abnormal circuit function found in ASD. We then identify emerging *in vivo* and *ex vivo* techniques, focusing on how these assays can characterize circuit level dysfunction and determine if these abnormalities underlie abnormal clinical electrophysiology. Such circuit level study in animal models may help us understand how diverse genetic and environmental risks can produce a common set of EEG, MEG and anatomical abnormalities found in ASD.

## The promise of translational phenotypes in ASD

Autism spectrum disorders (ASD) have an estimated prevalence of 1 in 68 children, potentially reaching as high as 1 in 42 males (Developmental Disabilities Monitoring Network Surveillance Year 2010 Principal Investigators; Centers for Disease Control and Prevention (CDC), 2014). Although the criterion for ASD has recently been updated, it is still considered a disorder of social impairments and restricted/repetitive behaviors (American Psychiatric Association, 2013). Findings of multiple weak or rare and often non-specific genetic or environmental etiologies of ASD have made it difficult to identify the common neurobiological mechanisms underlying behavioral features that define ASD. Despite this genetic and phenotypic heterogeneity, research over the last decade using Electroencephalography (EEG) and Magnetoencephalography (MEG) (E/MEG) has identified consistent differences in ASD electrophysiology, indicating common neural circuit disruptions (Wilson et al., 2007; Roberts et al., 2008; Gandal et al., 2010; Rojas et al., 2011; Edgar et al., 2013). Unfortunately the neuronal underpinnings of these electrophysiological biomarkers of ASD are not understood. The goal of this review is to describe emerging approaches in animal models to identify the circuit mechanisms that underlie these clinical E/MEG findings. To do so we first summarize the current literature of observed alterations to neural circuits in ASD, with a focus on mechanisms that may underlie the E/MEG phenotypes found in human subjects. Second, we propose that it is at the mesoscopic circuit level, particularly local circuit function that leads to high frequency activity, where many diverse alterations must integrate to produce the symptomatology of ASD. Finally, we highlight emerging techniques in assaying neural circuit abnormalities that may identify the clinical differences found in high frequency cortical activity. Thus we hope to identify how the basic science of oscillatory activity and connectivity in the brain, combined with known E/MEG phenotypes of ASD, can provide the basis for new testable hypotheses of ASD.

### The challenge of integrating multiple pathogenic mechanisms: no smoking gun

Studies over the last 40 years have revealed a multitude of alterations in ASD, ranging from genetic risk factors, to differences in whole brain connectivity (Pardo and Eberhart, 2007). As genetic screening began to investigate the inheritance profile of ASD, it was hypothesized that ASD may be operating on the polygenetic interaction of three genes, and that this interaction could be revealed in a sample of 60 pairs of ASD affected siblings (Piven, 1997). Within 8 years this estimate of underlying genetic profile of ASD had increased to involve 15 genes, along with strong indication of environmental factors (e.g., prenatal insults) (Santangelo and Tsatsanis, 2005). This trajectory of etiological complexity continues. Despite the presence of a disorder that impacts well over 1 in a 100 individuals, there is not a single gene or set of genes that strongly or exclusively generate ASD, and we have been unable to extrapolate any individual putative genetic deficits to neurological abnormalities that produce symptoms of ASD. Nevertheless, to produce the constellation of symptoms that define ASD, different genetic and other alterations involved in the production of ASD likely act further downstream to converge on common effectors that produces the symptomatology of ASD. Finding such a common factor at the neuronal level for ASD or related endophenotypes is a clear goal in the field of ASD research, yet success has been limited.

### Multiple synaptic receptors and neurotransmitters are affected in ASD, with no single coherent effect

Given this significant genetic heterogeneity, recent work has focused on identifying common networks and molecular pathways that may integrate multiple diverse disease risk factors (Parikshak et al., 2013). For example, a large number of ASD candidate genes are involved in developing, maintaining, or modulating synaptic connectivity. A genetic alteration repeatedly found in ASD is the duplication of the 15q11–13 locus, which is estimated to be presented in as high as 3% of the idiopathic ASD population (Meguro-Horike et al., 2011). This region is known to contain many genes that encode for GABA related proteins, such as postsynaptic GABA receptors. Separately, others have found that both GABA_A_ (Fatemi et al., 2009b) and GABA_B_ (Fatemi et al., 2009a) receptors levels are significantly decreased in postmortem brain samples from subjects with ASD. On the synthesis side of GABA signaling, GAD65 and 67 expression is decreased around 50% (Fatemi et al., 2002). Recent brain imaging studies have corroborated GABAergic post-mortem results, demonstrating decreased cortical GABA levels (Harada et al., 2011; Rojas et al., 2014), with some regional heterogeneity across the cortex (Gaetz et al., 2014).

γ-Aminobutyric acid signaling is not the only neurotransmitter system affected in ASD; glutamatergic signaling is also affected, with the expression of multiple genes and proteins regulating this signaling pathway (e.g., EAAT and AMPA isotypes) increased in cerebellar cortex of subjects with autism (Purcell et al., 2001). This finding of increased glutamatergic expression fits with recent *in vivo* findings using magnetic resonance spectroscopy (Brown et al., 2013), although the exact meaning of these results are unclear. Lastly the GluR6, a kainate receptor subunit, has been strongly linked to ASD risk (Jamain et al., 2002; Shuang et al., 2004; Strutz-Seebohm et al., 2006). This, when combined with glutamatergic phenotypes in mouse models that recapitulate key aspects of ASD, has led to hypotheses that altered glutamate transmission may contribute to the core phenotypes of ASD (Carlson, 2012).

As glutamate and GABA receptors comprise the majority of ligand-gated ion channels in the CNS, these genetic findings support the general neurophysiological hypothesis that a disruption of excitation/inhibition (E/I) balance contributes to the disorder (Rubenstein and Merzenich, 2003). Such proposed imbalance has been a common theme in multiple psychiatric and neurologic disorders, including Alzheimer’s disease, schizophrenia and epilepsy (Eichler and Meier, 2008). Yet, though there may be similarities between those disorders and ASD, there are differences in the development and symptoms of these diseases that must be explained by specific neurobiological mechanisms to explain the divergent symptoms. While this idea is limited in specificity; if E/I imbalance is one of the underlying principle components of ASD, an immediate consequence such an alteration would be perturbed circuit activity underlying oscillatory activity in the brain. Oscillations across the frequency spectrum are evidenced and theorized to be dependent on the strength and kinetics of inhibitory and excitatory synaptic interactions at the mesocircuit level (Buzsáki and Wang, 2012). While again, this is not specific to ASD, other disorders such as schizophrenia (Kwon et al., 1999) and bipolar disorder (Maharajh et al., 2007) also show alterations to oscillatory function. What may distinguish ASD is the exact circuitry involved, the developmental timing of the disruption or the set of neurophysiological abnormalities due to specific genetic and environmental factors. For instance, preclinical work has shown that altered high frequency activity due to E/I imbalance generated in prefrontal regions but not visual cortex, is sufficient for the production of social and fear-related, but not locomotive impairments (Yizhar et al., 2011). Furthermore, this was limited to specifically CAMKII but not PV expressing neurons. In humans, oscillatory activity in specific regions have been shown to scale with impairment measures of autism specific behaviors or social ability in various conditions including, response to auditory stimuli (Rojas et al., 2011), resting (Cornew et al., 2012) or prestimulus (Edgar et al., 2013). Specificity may come from the overlap of the genetic risks for multiple psychiatric disorders (Cross-Disorder Group of the Psychiatric Genomics Consortium; Genetic Risk Outcome of Psychosis (GROUP) Consortium, 2013). It is proposed that the genetic risks are not specific to disorders, but rather domains of symptomatology instead. For instance pre-frontal E/I imbalance, due to the genetic risks common to ASD and schizophrenia, may lead to social impairments that are shared between the disorders.

### Neuromodulators affected in ASD

Strengthening the concept that there are multiple synaptic paths to a common circuit dysregulation in ASD, is the evidence that neuromodulatory systems are also disrupted. Looking through the lens of E/I imbalance, neuromodulators can act at the synapse directly or on the firing properties of a cell to alter the strength, kinetics and firing probability of cells involved in circuit E/I balance and thus impacting neural functioning in ASD. In fact some of the earliest examined neurological abnormalities in ASD involved neuromodulatory systems (Boullin et al., 1970; Modahl et al., 1998), several of which are now known to be involved in emotional and social regulation. Peptide and monoaminergic transmitters appear aberrant in ASD, in particular the oxytocin and serotoninergic systems. These neuromodulators are of interest, because of oxytocin’s role in social behavior and bonding (Lieberwirth and Wang, 2014) partnered with a potential for treatment efficacy (Gordon et al., 2013; Tyzio et al., 2014), and the current use of pharmacological agents aimed at the serotoniergic system for treatment of ASD symptomatology. Both of these systems can impact cell excitability in an anatomical and cell-type specific manner. Oxytocin signaling is thought to be reduced in ASD (Modahl et al., 1998) and has been an intense focus for therapeutic intervention (Weisman et al., 2012; Gordon et al., 2013) and recent research at the circuit level (Owen et al., 2013). Individuals with ASD also exhibit hyperserotonemia, potentially arising from mutations in SLC6A4 and MAOA candidate genes (Harrington et al., 2013). The affect of the such genes may be two fold, serotonin plays an important role in the development of cortical and sub-cortical tissues, not only as a neurotransmitter but also earlier during development (Whitaker-Azmitia, 2001). The serotonin system remains altered in ASD during the juvenile period with an altered developmental trajectory of serotonin synthesis (Chugani et al., 1999). Interestingly in adults, if the levels of the precursor of serotonin is lowered via specific dietary manipulations, ASD symptomatology is exacerbated, again pointing to serotonin’s role in ASD (McDougle et al., 1996). This role of serotonin has been replicated in animal models, rats prenatally treated with 5-methoxytryptamine (5-MT), an agonist of serotonin receptors, demonstrate ASD like symptomatology and alterations to regions known to be involved in social activity and peptides release (such as oxytocin) (McNamara et al., 2008).

Acetylcholine (ACH) has also been focus of research with regards to pathology and treatment in ASD. Multiple studies show altered ACH related findings, such as decreased Positron emission tomography (PET) binding, altered post mortem immunoreactivity for receptors and mRNA, and decreased relative choline levels in *in vivo* in ASD (Deutsch et al., 2010). Interestingly, increasing levels of ACH specifically in striatal regions allows for the recovery of social and cognitive flexibility in an animal model that is thought to recapitulate those aspects of ASD (Karvat and Kimchi, 2014).

Another major neuromodulator linked to ASD is dopamine. Several studies have shown that dopamine is increase in frontal cortex (Chugani, 2012), and multiple genetic studies have linked dopamine associated genes with ASD (Nguyen et al., 2014). Consistent for a role of dopamine signaling in ASD, is their importance in mediating repetitive and stereotyped behaviors. Both from human genetic findings (Staal, 2014) and mice models (Chartoff et al., 2001). While oxytocin continues to be examined as a potential treatment, current drugs targeting these and other systems have not been found to be effective in treating core symptoms of ASD (Warren et al., 2011), suggesting that these modulatory system contribute to, but do not drive the disorder.

This non-exhaustive summary of alterations to the neurotransmitter and neuro-modulator systems suggests targets for treatment, yet also indicate that no single target will be broadly effective. Single perturbations are not consistently present throughout all cases of ASD, and furthermore such alterations are usually found in differing combinations; yet ultimately they synergize to produce the symptomatology known as ASD. Thus, hypothesis such as E/I balance suggests common neuronal circuit differences that could be targeted to repair circuit function in ASD.

### Immune system dysfunction

Maternal infections, fever and antibiotic treatment, as well as post-natal infections, are associated with an increased likelihood of the child being diagnosed with ASD (Hsiao, 2013). This relationship can be modeled in animals via injections of various components of pathogens, which lead to the discovery that the immune response driven by cytokines that can produce this link (Garay and McAllister, 2010). Of note, is the evidence from several studies that autoimmune diseases and/or allergies are at a greater prevalence in ASD (Hsiao, 2013). In fact it has been demonstrated that maternal antibodies reactive to fetal tissue are linked to ASD (Fox et al., 2012). These brain targeting antibodies also are found in patients with ASD, and certain sub-types correlate with specific ASD behavioral symptoms (Goines et al., 2011). At the cellular level, evidence of immune system involvement is found in ASD, with increased activation and number of migroglial cells both in patients with ASD and animal models that recapitulate ASD via genetic or environmental insults (Hsiao, 2013). The major histocompatibility complex (MHC) locus, a region of the genetic code which contains genes for immune system functioning, is of particular note to circuit function in ASD. Multiple genes and haplotypes in the MHC local are linked to higher incidence of autism (Needleman and McAllister, 2012). These MHC genes are important for neuronal development, connectivity, and circuit function (see Section Cellular Assessment of Circuit Activity: Targeting the Neurophysiology of Local Circuit Dysfunction in ASD).

### Structural and connectivity alterations are present in ASD

Sub-cellular and synaptic alterations are not the only alterations found in ASD. At the level of local cytoarchitecture, post-mortem studies of individuals with ASD have shown increases in the density of minicolumns in multiple cortical regions, while each column itself is narrower, primarily due to decreases in peripheral neuropil compartments (Casanova et al., 2010). These cytoarchitectural changes are in concert with increased rates of macrocephaly in individuals with ASD, for which increases in brain volume are disproportionately greater for local cortical white matter connections (Herbert, 2005). Such local increases in anatomical connectivity, observed in ASD as an increase in local white matter tracts, may exist alongside decreases in long-range connectivity (Jou et al., 2011). Of note, alterations in long range connectivity have been correlated with electrophysiological deficits in bottom-up sensory processing (Roberts et al., 2009). Thus, ASD are associated with alterations across function, modulation, and structure. While there are likely causal relationships between alteration in any of these components (activity during development leading to structural changes or vice versa), the heterogeneity of any of these findings within and outside ASD also suggest that they can combine to produce a common set of neurological abnormalities. Assaying the combined and potentially differing impact of these diverse potential etiologies is limited in human patients and technically difficult in model systems.

### EEG and MEG studies present common clinical neurophysiological differences in ASD

**Figure 1:**
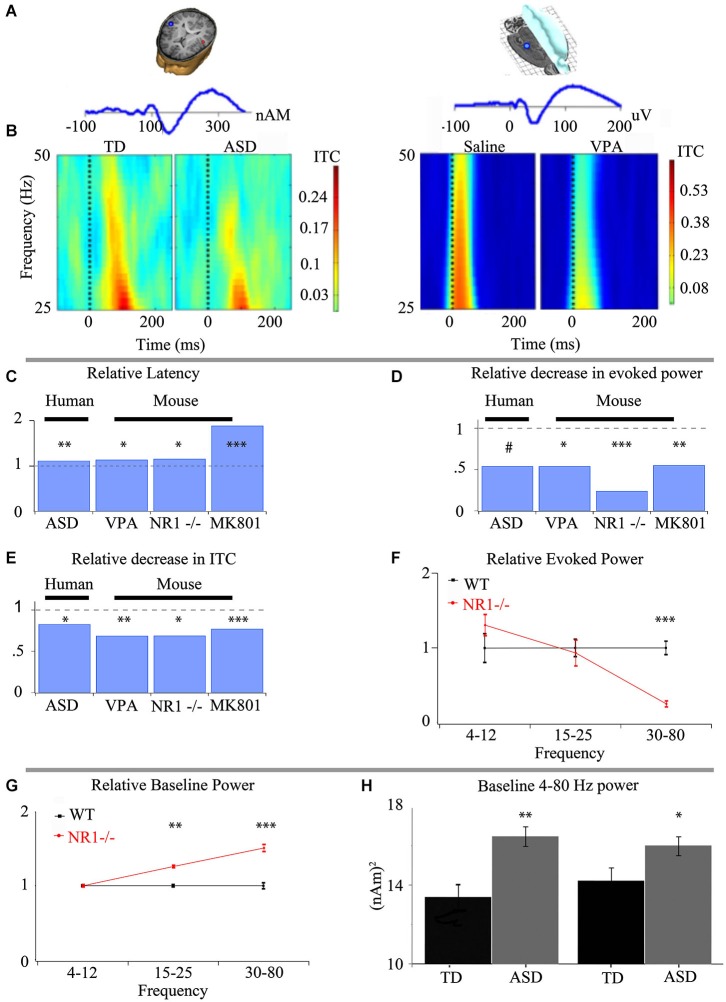
**Intermediate phenotypes seen in human subjects are translatable to animal models that recapitulate differing aspects of ASD**. **(A)** Representative dipole/electrode placement (top) and ERP trace (bottom) for control human (left) and mouse (right) appear qualitatively similar. **(B)** Time-frequency plots of phase-locking factor for humans (left panel) and mouse (right panel) exhibit direct translatability. Note the decrease in phase locking in gamma frequencies in both ASD and an environmental insult based model of ASD. **(C)** Event-related potential latencies are delayed both in children with ASD and animal models of ASD utilizing prenatal insult, genetic insult and pharmacological treatment. Similar deficits in gamma-band responses are seen between children with ASD and numerous animal models of ASD with varying biological mechanisms for both evoked power **(D)** and inter-trial coherence/phase-locking factor (ITC/PLF) **(E)**. **(F)** The observed alterations to the spectral response are specific to gamma-band frequencies when examined in an animal model recapitulating keep aspects of ASD. **(G)** As proof of the predictive validity of translational electrophysiology, increases in baseline activity, first observed in animal models utilizing a genetic basis **(G)**, have been demonstrated in children with ASD **(H)**. *** *P* < 0.001, ** *P* < 0.01, * *P* < 0.05, # *P* < 0.1 Figure adapted from Gandal et al. (2010)—**(A–E)**, Gandal et al. (2012a)—**(C–E)**, Gandal et al. (2012c)—**(C–F)**, Saunders et al. (2012)—**(C–E)**; **(H)** courtesy of J.C. Edgar, adapted from Edgar et al. (2013).

In contrast to the divergent assortment of complex combinatorial risks found for ASD, clinical electrophysiology has identified specific resting, event related potential, and spectral changes that suggest common neural circuit function abnormalities (Figure 1). In particular spectro-temporal processing of auditory stimuli in ASD demonstrates deficits in gamma-band activity (30–50 Hz) (Wilson et al., 2007; Rojas et al., 2008; Gandal et al., 2010; Edgar et al., 2013). These alterations in stimulus-produced responses occur alongside aberrant resting state profile in ASD (Orekhova et al., 2007; Cornew et al., 2012). As such, coherent alterations have been identified in ASD, which point to common neurobiological underpinning. Because of the complexity of the neurophysiology underlying these clinical electrophysiological findings, there are many points where differing etiologies could perturb these measures. This poses the critical question: How is the ensemble neuronal activity that generates the EEG and MEG signals perturbed in ASD?

### ASD related E/MEG endophenotypes are replicated in multiple models related to ASD and point to local circuit change

The alterations detected via E/MEG in auditory event related potentials and gamma-band abnormalities have been a focus of translational research, since auditory processing in mice, rats and humans is remarkably well conserved (Figure 1). Event related potentials themselves have shown an increased latency in both MEG and EEG in ASD and representative animal models respectively. These findings indicate differences in the speed of auditory processing as information is passed via auditory nuclei to the cortex (Figures 1A,C). Auditory responses to stimuli can also be separated into their spectral subcomponents, which exhibit a strong phase-locked gamma band activity between 30–50 Hz (Figures 1B,E). This ability of a subject to produce an alignment of gamma-band phases over multiple trials with respect to the stimuli can be viewed as the local circuit’s reliability. The less able the system is to respond to an input with an appropriate and controlled response, the more dysfunctional the system will be. This is especially important when the responses are basic sensory responses that need to be combined into meaningful stimuli. This quality of phase-locked activity between trials (reflected as ITC in Figure 1) is decreased in ASD individuals compared to typically developing age-matched subjects (TD). This finding has been replicated across research groups (Rojas et al., 2008; Gandal et al., 2010). Similarly, evoked gamma-band power in response to analogous auditory stimuli is also decreased in ASD, and animal models that recapitulate key phenotypes of ASD (Figure 1D; Gandal et al., 2010, 2012a; Saunders et al., 2012), suggesting neuronal mechanisms are shared between ASD and these models of the disorder. While ITC and evoked power are very similar entities, they remain crucially separate (Box 1).

Box 1 **ITC vs. evoked power, similar yet not redundant.**

Changes seen in ITC and evoked activity are related, since both are referring to phase and time locked activity, though ITC is questioning “what fraction of signals are in phase”, and evoked power asks “of those time and phase locked, what is the amplitude of the response”. If there are no in-phase signals then ITC would equal 0, and evoked power would also be 0. If responses were perfectly in phase ITC would equal 1 (its maximum possible value), and evoked power would be the amplitude of the resulting wave. Yet, because ITC is only a measure of trial-to-trial phase reliability and not the amplitude of power generated by the stimulus, ITC can be independently disrupted. Such a case could occur when a manipulation could increase the ability cells to more temporally accurately fire, but the total amount of cells firing is reduced.

In contrast to the loss of phase reliability across repeated stimuli, other findings in the gamma band are an increase in baseline spectral activity in ASD and increased stimulus related non-phase locked activity (often called “induced” activity (Wilson et al., 2007; Edgar et al., 2013 respectively)). Increases in gamma activity related to an event or stimuli that is not phase locked has traditionally been presumed to be involved in higher cognitive processing of the signal (Tallon-Baudry and Bertrand, 1999), but a feature in ASD is that the increase in induced activity appears coupled to a reduction in evoked activity, suggesting that in ASD there is a reduction in the ability of the brain to respond reliably to a event/stimulus and it is this loss phase-locked activity that may be increasing induced activity (Wilson et al., 2007).

From the multiple putative neuronal differences indicated in ASD it should not be surprising that gamma-band activity is impacted. The generation of high frequency oscillatory activity during ensemble activity requires low latency feedback between inhibitory and excitatory neurons, in the case of 40 Hz activity one cycle of activity must comprise 25 ms. Gamma-band activity is also sensitive to behavioral states and their associated neuromodulators (Schadow et al., 2009; Kim et al., 2014). Finally, unlike the event related potential latencies, changes in gamma-frequency may be due primarily to local changes in circuit connectivity (Kopell et al., 2000; Cardin et al., 2009; Wang and Carlén, 2012). Because of gamma’s dependence on local circuit function, many mechanisms of gamma can be assayed in brain slices. Thus, focusing on gamma-band changes in ASD and rodent models that share EEG validity with ASD (Gandal et al., 2010, 2012a,c; Saunders et al., 2012) may provide a path to directly assay mechanisms of gamma-band dysfunction using the enhanced access of *in vitro* preparations.

### Promise of circuit level focused examination of ASD: potential for translation between model systems and inquiry at multiple scales

Mesoscopic level examination of circuits, and in particular micro-circuits, maybe be the ideal area for investigation, because they integrate multiple sub-cellular to systems alterations. Not only is ensemble activity sensitive to genetic (Carlson et al., 2011), pharmacological (Saunders et al., 2012) and cellular manipulation (Cardin et al., 2009; Sohal et al., 2009; Billingslea et al., 2014), but it can provide insight into the human pathogenic markers of ASD. Identifying an intermediate phenotype at the population activity level may allow the translational bridging of basic, pre-clinical, and human-subject research. Furthermore it provides additional bridging of the molecular to behavioral domains.

Differences in circuit function activity in model systems may be easier to interpret in terms of changes in clinical electrophysiology found in EEG. There has been success modeling social and behavioral deficits as markers of ASD-like symptomatology (Silverman et al., 2010), yet questions remain of how to fully model and validate the behavioral disturbances seen in ASD and reliably link them with neurophysiological differences. Recapitulating ensemble electrophysiological activity that is seen in patients with ASD can provide mechanistic bridge between the construct validity of a model and its behavioral phenotypes. Gandal et al. (2010) demonstrated that the electrophysiological abnormalities seen in children with ASD were recapitulated in a prenatal insult based mouse model of ASD (Figure 1), and these changes correlated with synaptic protein expression and behavior. Similarly analogous changes have been seen in response to pharmacological treatment (Saunders et al., 2012) and genetic manipulations (Gandal et al., 2012a). Thus, with increasing evidence in animal models for recapitulation of EMEG phenotypes of ASD it becomes important to ask what are the underlying neurophysiological differences that mediate observed changes in cortical ensemble activity that in turn lead to disrupted E/MEG activity in ASD.

To make the link between circuit function and E/MEG differences three areas of inquiry show promise: (1) direct cellular modulation of local circuit properties; (2) assays of ensemble activity linking circuit dysfunction to E/MEG phenotypes; and (3) measures of functional connectivity. Below we discuss each of these approaches.

## Current circuit level research in ASD: current methodologies for future advances

### Cellular assessment of circuit activity: targeting the neurophysiology of local circuit dysfunction in ASD

**Figure 2:**
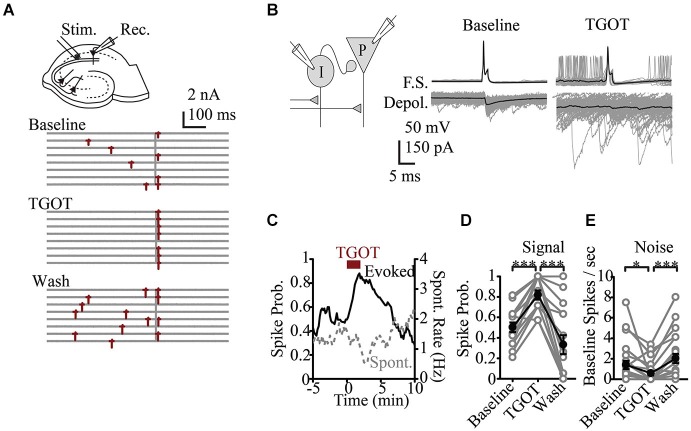
**Effects of oxytocin at the circuit level**. **(A)** Representative response to Shaffer Collateral stimulation (vertical bar) of a CA1 pyramidal cell (red—spike, TGOT—Thr^4^,Gly^7^-oxytocin—specific agonist for oxytocin receptors) **(B)** Responses of presynaptic interneurons (gray—sweeps, black—average) to TGOT, note effect is to increase baseline and reduce stimulus evoked firing. **(C)** Time course of spike probability for both evoked spikes (black) and spontaneous events (gray) from pyramidal neurons. Spike probability in response to stimulation **(D)** and at baseline **(E)**. Figure courtesy of R.W. Tsien, adapted from Owen et al. (2013). *** *P* < 0.001, ** *P* < 0.01, * *P* < 0.05, # *P* < 0.1.

Recent basic science research at the microcircuit level has had particular relevance to ASD. While there is a wealth of data looking at synaptic or cellular firing properties in putative models of autism, there are few studies that directly assay modulation of circuit activity. An exception is *in vitro* work in the hippocampus that looked at the role of the peptide Oxytocin. Oxytocin, is a neuromodulatory peptide known to be important in social bonding, prosocial behavior and social recognition (Lieberwirth and Wang, 2014) and is reduced in patients with ASD (Modahl et al., 1998), making it an area of interest regarding therapeutics (Weisman et al., 2012; Gordon et al., 2013; Tyzio et al., 2014). At the single cell level Oxytocin was known to increase excitability (Raggenbass et al., 1989). It was therefore surprising that in response to a compound synaptic potential, where both local inhibitory and principle cells are activated by synaptic input, oxytocin signaling was shown to regulate the fidelity of stimulus produced firing and reducing baseline neural activity (Owen et al., 2013). This was because the most prominent affect of oxytocin was to increase spontaneous activity in the inhibitory neurons and thus increase baseline GABA related inhibitory tone of the system. By increasing spontaneous inhibitory activity, the feedforward inhibitory signaling evoked by afferent stimuli was reduced. Thus a more faithful transmission of signaling and better signal-to-noise ratio was achieved (Owen et al., 2013; Figure 2). Such increase in reliability may scale at the ensemble level to produce a greater trial-to-trial phase reliability detected in EEG or MEG signal that is measured as increased ITC. Analysis at this cellular circuit level in models relevant to ASD may similarly identify underlying changes in reliability that could be linked to reduced ITC in patients. Importantly, such a role for oxytocin would be opaque, without studying the interactions of excitatory and inhibitory cells within their microcircuit context.

Studying microcircuits can also be used to study changes in the number or strength of synaptic interconnections. Using a model of prenatal exposure to the antiepileptic drug valproic acid, which is associated with a ~7 fold increased risk for autism, Rinaldi et al. (2008) patch-clamped multiple connected cells in cortical slices to measure connectivity between neurons. Consistent with interpretations of hyper local connectivity from post-mortem anatomical data, in valproic exposed animals they showed local circuit hyper-connectivity and hyperactive cortical local networks.

Mesoscopic level dysfunction can also be detected due to immune system based insults. Alterations to the retinogeniculate pathways, as well as more local cytoarchitectural alterations in hippocampus, cortex and retina, are well documented in mice with MHC class I proteins perturbations (Elmer and McAllister, 2012). Such architectural modifications are not the only effect MHC class I related alterations, perturbations to in circuit function with regards to plasticity, and critical periods in several animal models also exist (Boulanger, 2009). These changes in plasticity can arise from changes to the intrinsic cellular properties of neurons, such as increases in excitability and frequency of mEPSCs (Boulanger, 2009). Following this trend, mice with MHC related alterations can show altered E/I balance in cortex and short term plasticity modifications in cerebellum (Elmer and McAllister, 2012). Plasticity over the longer term is also aberrant in these animal models were altered LTD/LTP is exhibited (Elmer and McAllister, 2012).

Using monogenetic insults that mimic syndromes similar to ASD, plasticity has also been found to be perturbed in multiple animal models. In a mouse model of fragile X disorder, a disorder closely linked to ASD, mice demonstrated increased LTP (Huber et al., 2002). Plasticity alterations have been found in an model of Angelman syndrome, where again LTP and LTD were perturbed (Yashiro et al., 2009). Moreover, these mice also demonstrated altered visual cortex plasticity with decrease ocular dominance following monocular derivation (Yashiro et al., 2009). Alterations to visual cortex plasticity, was also demonstrated in a mouse model of Rett’s syndrome, with a MeCP2 mutation extending the period within which alterations could occur (Tropea et al., 2009). Interestingly, when traditional measures of spine phenotypes and protein production were recovered so was ocular dominance in a similar model of fragile X as used by Huber et al. (2002) and Dölen et al. (2007). Such ocular dominance can be measured as an ensemble activity by analyzing visual event related potentials. This demonstrates both monogenetic models and immune system dysfunction can produce measured circuit level dysfunction, and therefore may have promise in identifying common features of circuit related disorder in ASD.

### Ensemble activity

Ultimately, to produce changes measured at an EEG electrode, micro-circuit changes need to be expressed by large numbers of neurons producing ensemble activity. Using voltage sensitive dye imaging (VSDi), that can measure voltage changes across all the excitable membrane (Box 2), this ensemble activity can be studied directly in slices. Comparable changes as predicted by cellular studies, have been found in other models of ASD and ASD-related syndromes. For instance mice lacking MeCP2, which model Rett Syndrome, have been shown to be hyperexcitable, and possibly hyperconnected, within hippocampal circuits using VSDi, shedding light on previous dichotomous results of single cell recordings (Calfa et al., 2011).

Box 2 ***In vitro* techniques to study spatial and temporal qualities of cortical gamma-band abnormalities.**

**Figure 3:**
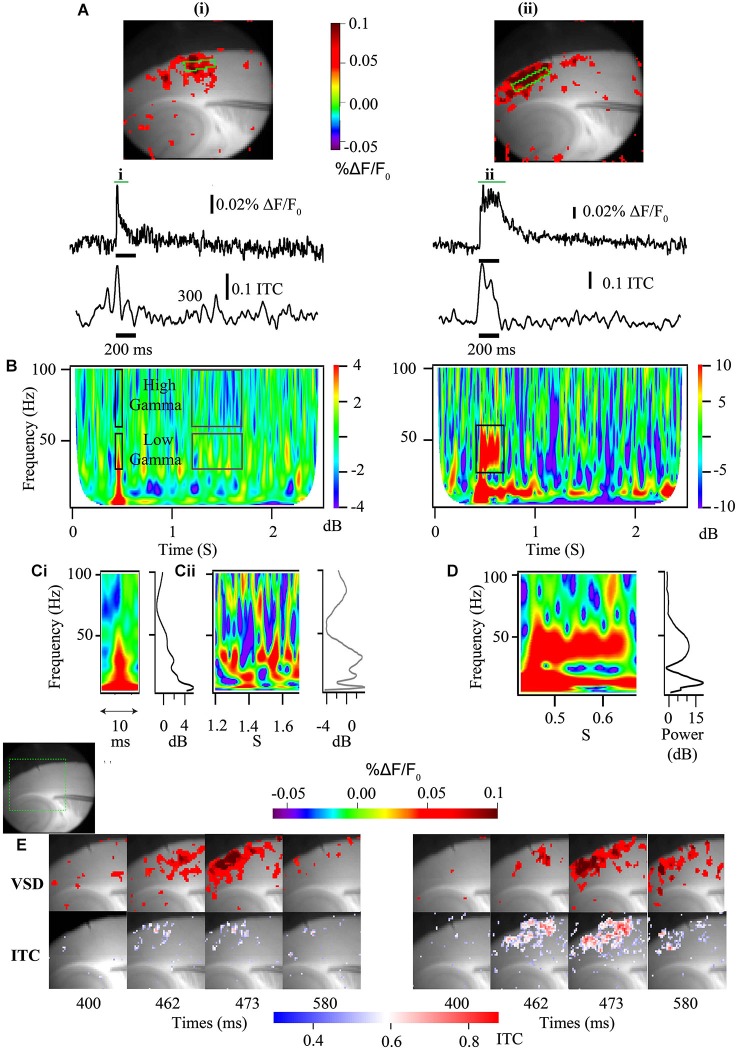
**Voltage sensitive dye imaging signals are amenable to time-frequency decomposition. (A)** Auditory cortex VSDi responses to single stimulation (left) and burst stimulation at 40 Hz of white matter input tracts (right). Top—2D representation of evoked response [green square—ROIs used below]. Middle—VSDi florescence traces of the ROI depicted in above for single stimulation (left) and burst stimulation (right). Bottom—30–50 Hz ITC calculated for same ROI. **(B)** Time frequency plots for single stimulation (left) and burst stimulation (right). Note that the single stimulation time frequency plot can be separated not only into high and low gamma, but also stimulus related (black boxes) and baseline activity (gray boxes). For the burst stimulated time frequency plot a large increase in power occurs around the frequency that is stimulated at (black box). **(C)** Parsed stimulus related activity **(i)** and baseline activity **(ii)** for the single stimulation paradigm demonstrated in **(B)** [left—time frequency plot, right—image profile of time frequency plot]. Note local peak of activity at 40 Hz in image line profile for both stimulus related activity and baseline. **(D)** Parsed period of stimulation [left—time frequency plot, right—image profile of time frequency plot] for the 40 Hz burst stimulation paradigm demonstrated in **(B)**. Note large increase in 40 Hz power. **(E)** Series of stills from a region of interest (green dashed box in gray scale image shows relative location) in response to a single stimulation (left) and a 40 Hz burst stimulation (right) [top frames—standard VSDi imaging measure; bottom frames—35–55 Hz band passed movie from which pixel-wise ITC has been calculated and overlaid back onto grayscale image]. Note that this analysis allows the determination of laminar and regional existence of inter-trial coherence (ITC).

Voltage sensitive dye imaging allows for the direct assay of membrane voltage. Because of most of the excitable membrane available for VSDI in the brain is found in dendrites (for review see Chemla and Chavane, 2010) the VSDI signals are biased towards the membrane that is most involved in generating the cortical cellular dipoles recorded at the MEG or EEG sensors (Buzsáki et al., 2012). When used to study the population level, the summed membrane responses can measure the kinetics of the membrane response and this can be associated with differences in EEG responses at the *in vivo* level (Carlson et al., 2011) as well as being amenable to direct examination via time frequency analysis (Figure 3).

Similar to how sensory-elicited changes in cortical population responses are reflected in E/MEG power (Figure 1), *in vitro* studies can use the population responses driven by electrically evoked afferent activity to more directly study the interplay between excitatory input and the coupled oscillations of excitatory and inhibitory neurons. In areas such as the neocortex and area CA3, which support spontaneous population gamma events *in vitro* (Köhling et al., 2000; Csicsvari et al., 2003; Cunningham et al., 2004), afferent stimulation will generate an increase in membrane potential as well as power when frequency is analyzed (Figure 3; Prechtl et al., 1997). Thus, these more complex local interactions can reveal the same time-frequency components measured from repeated sensory stimulation in human subjects, including evoked, ITC and induced power, as well as the spectral background population activity. In EEG/MEG these different components can be mapped to specific dipoles. When these components are identified *in vitro* using VSDi, they also can similarly be mapped back to specific lamina and the extent of functional coupling among horizontal components measured (Figure 3). This level of laminar specificity may be important for identifying the cortical circuit components that are disrupted in ASD. In auditory cortex it has been demonstrated that higher frequency gamma oscillations (50–80 Hz) occur primarily in layer 4, with a high dependence on NMDA signaling (Ainsworth et al., 2011). On the other hand lower frequencies of gamma oscillations (30–45 Hz) arose from layer 2 and 3 are highly reliant on gap junctions (Ainsworth et al., 2011). Using cortical slice preparations from validated models of EEG phenotypes, it appears possible to map E/MEG endophenotypes of ASD to specific lamina (Figure 3E). As these laminae are characterized by specific developmental origin, cell types and connectivity, identifying the laminae involved in producing specific EEG phenotypes can help detect the developmental and cellular etiology underlying the E/MEG phenotypes.

While it appears that circuit-level study of animal models that recapitulate key aspects of ASD are finding common alterations, there has yet to be one study or set of studies that takes one model of ASD and examines it from the cellular through to the EEG level, thus directly linking the cellular to the ensemble and on to intermediate phenotypes and behavior.

While experiments *in vitro* using animal models that recreate key aspects of ASD have demonstrated promise, work targeting ensemble activity that generate abnormal oscillatory activity in ASD could provide vital cues. By extrapolating the interaction of known cellular components and their connectivity, and modeling them as circuits *in silico*, pioneers in the field of neuronal oscillations made great strides in identifying the types of circuit activity that can generate specific patterns of oscillations recorded by EEG. Original *in vitro* work combined field, intracellular recordings and modeling to identify the neurophysiological elements that support such ensemble activity (Traub et al., 1996, 1999; Ermentrout and Kopell, 1998). These groups identified intracellular and specific cellular qualities that could generate gamma-band activity, which included potassium channel subtypes, spike conductance trajectories and a strong role for both glutamatergic and GABAergic signaling (Buzsáki and Wang, 2012). One repeated finding was that gamma-band activity occurring around 40 Hz band is strongly dependent on GABA receptor activity (Whittington et al., 2000). Thus, in diseases much of the focus on gamma-band abnormalities has been the role of inhibition.

Studying gamma band activity at the local ensemble level takes advantage of the fact that gamma-band (30–100 Hz) activity measured by E/MEG are produced by synchronous neuronal activity arising from these local cortical interactions (Buzsáki and Wang, 2012; Buzsáki et al., 2012). By assaying and modulating ensemble activity, researchers have been able to identify the cell types involved and begin to validate their impact on EEG using modern measures and modulation of ensemble activity. As such the E/MEG detected phenotypes of ASD can be teased apart, potentially leady to a common substrate.

### Targeting specific cellular components to understand their impact on ensemble activity

Post-mortem studies and transgenic models where genetic changes are limited to specific cell types anatomical areas have suggested that changes primarily limited specific cell types and brain areas may underlie ASD related symptoms and clinical electrophysiological abnormalities. Using optogenetics to optically excite or inhibit specific cell types involved in oscillatory activity has shown great promise to directly test these hypotheses. The prominent role of parvalbumin (PV) fast spiking cells in generating high-frequency oscillations was demonstrated by specifically expressing the photoactivated Cl^−^ pump halorhodopsin *in vivo* (Sohal et al., 2009). The reduction in gamma-band activity when PV cells were disabled is consistent with the role of GABA receptors in high-frequency activity. This study was in line with evidence of the role of PV cells in disorders such as schizophrenia, which demonstrate both disruptions in PV cell immunoreactivity (Zhang and Reynolds, 2002) and gamma-band function (Sun et al., 2011). Optogenetics has also been used to link *in vivo* cortical E/I balance, gamma-band oscillations and social behavior by directly increasing excitation in the medial prefrontal cortex (Yizhar et al., 2011). Though there are limited indications that PV cells are disrupted in autism (Lawrence et al., 2010) connectivity involving these and other inhibitory cells types may be abnormal, which may lead to multiple changes in cell type excitability (Zikopoulos and Barbas, 2013). The problem therefore remains that within ASD, differing cell types, synaptic disruptions or connectivity may contribute to similar oscillatory dysfunction. Because optogenetics can be used to excite and inhibit different genetically targeted cell types or anatomical areas, it is well suited to probe the differential contributions of cellular and synaptic dysfunction that may lead to changes in oscillatory activity. In particular where the specific disruption in the E/I balance or connectivity may lead to increased excitation in one cell type and reduced excitation in another, the use of ontogenetic probes with different excitation wavelengths and kinetics can potentially mimic or more fully reverse those conditions (Yizhar et al., 2011).

### Assessing functional connectivity within the cortex

Circuit based findings could also aid in testing other hypotheses of ASD. Connectivity, and in particular regulation of functional connectivity is very apt to be studied using circuit/ensemble based techniques as demonstrated in Wilson et al. (2012). Moreover Gray et al. (1989) demonstrated the ability to examine ensemble functional connectivity within cortex, akin to what is detected with EEG but with more precision. Here recording sites located in non-overlapping receptive fields of visual cortex with similar preferred orientation, synchronized maximally for a stimuli that were continuous between recording site’s respective receptive fields. Ensemble functional connectivity remains of consequence because along with changes in cellular and receptor properties, alterations in cortical connectivity have been reported in subjects with ASD (Belmonte et al., 2004). High-density electrode arrays or VSDI recordings *in vitro* from cortical slices and *in vivo* from cortical surface can be used to assay the functional connectivity that is associated with local coherence in E/MEG. An immediate area of investigation could directly identify how connectivity is mediated (e.g., directly via axon connectivity or via unmasking of surround inhibition), using *in vitro* pharmacological tools and differing ionic composition (Ang et al., 2005). Ultimately it could provide functional analysis of layer specific connectivity; while directly imaging the surface *in vivo* can precisely map the extent of activity in animals modeling ASD.

Changes in functional connectivity can be incredibly sensitive to alterations to even molecular constituents. For example knocking out the potassium channel KV3.2 in mice reduces high frequency, but not low frequency correlated activity within cortical locations. In these KV3.2 −/− mice, the lack of high frequency coherence was not due to single site reductions in high frequency power, which were unchanged from wild type counterparts, but alterations in coherence (Harvey et al., 2012). Such changes to coherence have the potential to cause behavior abnormalities in *in vivo* systems. Operant learning has been shown to be coincident with increase in coherence between striatum and amygdala via coupling in the high frequency range (Popescu et al., 2009). While anxiety has been associated with increase in hippocampal synchrony between the hippocampus and prefrontal cortex at theta frequency (Adhikari et al., 2011). Such specificity of high-frequency coherence may be opaque to imaging modalities with lower temporal resolution, such as fMRI, or less spatial resolution for deep structures, such as E/MEG. Thus, preclinical models may be the only efficient way to studying disease specific disruption of such functional connectivity between coupled nuclei at higher frequencies.

### Pre-clinical EEG and LFP recordings

*In vivo* population recording in mice and rats obtained using implanted electrodes to measure EEG also provide useful circuit based insights into ASD. The ability to detect analogous alterations in population activity as seen in ASD was demonstrated with the prenatal VPA insult model of ASD as well through genetic reduction in NMDA receptors (Figure 1; Gandal et al., 2010, 2012a). The behavioral deficits seen in this NR1 reduction model correlate with the observed increase in gamma-band power (Gandal et al., 2012c). More importantly, in this model reduction of ASD-like symptomatology was mirrored by a rectification of gamma-band activity (Gandal et al., 2012c). Such changes in baseline power prompted the examination of clinical data, where similar increases in power was found in children with ASD (Edgar et al., 2013), demonstrating directly the important of translational electrophysiology. Similar techniques are now being combined with cell-selective receptor manipulations, producing differing phenotypes, both for behavior and ensemble activity recordings (Billingslea et al., 2014). Such an approach may be useful in other models where cell or location specific genetic modifications have led to ASD related phenotypes.

### Current limitations of this approach

Focusing on circuit level analysis of ASD pathogenic mechanisms does have its own weaknesses. For example findings of delays and reduced dynamic range in electrophysiological markers of auditory processing (M100) in ASD have been well documented (Roberts et al., 2008; Figure 1). Currently such phenomena are difficult for circuit level analysis to dissect as it likely involves complex system level interactions that are difficult to assay. Nevertheless, recent improvements in technique such as multielectrode recordings in rodent cortex has allowed investigators to tease out different onset latencies in auditory fields and coherence between auditory fields (Centanni et al., 2013). Such work is showing promise at illuminating the regional electrophysiological dysfunctions within the auditory fields associated with an environmental insult thought to model ASD in rodents (Engineer et al., 2014).

Another important challenge for the circuit level approach is to determine the exact role that the observed electrophysiological abnormalities play in the specific symptomatology of ASD, whether they are specific to core symptoms or more cognitive issues. This is of importance because of the appearance of similar electrophysiological findings in disorders such as schizophrenia (Uhlhaas and Singer, 2006; Gandal et al., 2012b). In schizophrenia, gamma-band alterations are correlated to negative symptomatology and treatment resistant symptoms, and have to be shown to mirror improved cognitive activity after pharmacological treatments (Gandal et al., 2012b). The exact relation of local circuit activity to behavioral outcomes across multiple disorders must be established.

The question of specificity of electrophysiological findings with regards to ASD symptomatology also arises since the aforementioned markers, such as disrupted gamma-band activity, have been found in first degree relative of patients with ASD (Rojas et al., 2008, 2011; McFadden et al., 2012). First-degree relatives of patients with ASD often exhibit a broad autism phenotype however (Losh et al., 2008), and as such may reflect this behavioral predisposition. This leads to hypothesis that electrophysiological makers such as M100 delay and gamma-band dysfunction may signal a risk for ASD or schizophrenia rather than be a sign of the disease, thus more clinical work is needed to better understand the role of high-frequency changes in activity associated with ASD. Nevertheless, this does not limit the importance of understanding the circuit mechanisms underlying these common E/MEG phenomena, rather it suggest care in interpretation and a need to dissect the connection between disordered circuit activity and behavior. Conversely, recovery of normal gamma-function appears to predict amelioration of behavioral deficits in some models of ASD (Gandal et al., 2012c), and inducing higher baseline gamma-band activity can inhibit social exploration (Yizhar et al., 2011). Such studies have not been completed in ASD patients. If such a link between E/MEG and treatment efficacy is established in patients, the significance of such circuit level studies will increase significantly.

## Summary: circuit-level examination shows potential for unifying the current state of ASD findings

Synaptic dysfunction, local and long range alterations in connectivity, as well as aberrant modulation can each contribute to altered circuit function, leading to abnormal ensemble activity and disordered brain function. Such changes in brain function likely result in the symptoms of ASD. These changes in ensemble activity and brain function are reflected in differences in EEG, MEG and other functional imaging modalities, yet the actual neurophysiologic mechanisms remain to be identified. The current and developing techniques available to investigators finally allows for the examination of circuit related alterations in ASD, which is critical to bridging cellular and molecular changes commonly studied in animal models and recent clinical electrophysiology from patients with ASD. Circuits that produce gamma-oscillations appear sensitive to changes ranging from subcellular expression to white matter tract alterations. These circuit abnormalities also appear to be a locus where different ASD-related markers produce similar effects, thus the ASD related gamma changes are a fruitful area for translational inquiry. For instance gamma-band activity has been correlated to hallmark behavioral phenotypes (Rojas et al., 2011), and translational research has demonstrated behavioral improvement due to pharmacological treatment can mirror amelioration of gamma-band function in preclinical models (Gandal et al., 2012c), indicating such an approach can help lead to better treatments in human patients.

Other psychiatric disorders (ADHD and schizophrenia) have similar genetic underpinnings with ASD (Cross-Disorder Group of the Psychiatric Genomics Consortium; Genetic Risk Outcome of Psychosis (GROUP) Consortium, 2013). There is also similar changes in resting and evoked gamma, and a differential impact on EEG coherence between and within hemispheres in these disorders (Uhlhaas and Singer, 2006). Thus, the study of circuit dysfunction in ASD may refine the unique or common cellular character of the E/I imbalance and connectivity seen in multiple disorders. In particular further developing these measures in models and patients can provide the basis for testing the interaction of specific circuit abnormalities and cognitive and behavioral domains associated with these diseases as codified in the Research Domain Criteria by the NIMH (RDoC). As such these developing measures may provide the necessary neurophysiological foundation for understanding differences within ASD and between ASD and other psychiatric disorders. By identifying these mechanisms we can design interventions that directly target circuit function.

## Authors and contributions

All authors contributed to the preparation and finalizing of this manuscript.

## Conflict of interest statement

The authors declare that the research was conducted in the absence of any commercial or financial relationships that could be construed as a potential conflict of interest.
